# A Triangle Mesh Standardization Method Based on Particle Swarm Optimization

**DOI:** 10.1371/journal.pone.0160657

**Published:** 2016-08-10

**Authors:** Wuli Wang, Liming Duan, Yang Bai, Haoyu Wang, Hui Shao, Siyang Zhong

**Affiliations:** 1 College of Mechanical Engineering, Chongqing University, Chongqing, China; 2 Engineering Research Center of Industrial Computed Tomography Nondestructive Testing of the Education Ministry of China, Chongqing University, Chongqing, China; 3 College of Information and Control Engineering, China University of Petroleum, Qingdao, China; Beihang University, CHINA

## Abstract

To enhance the triangle quality of a reconstructed triangle mesh, a novel triangle mesh standardization method based on particle swarm optimization (PSO) is proposed. First, each vertex of the mesh and its first order vertices are fitted to a cubic curve surface by using least square method. Additionally, based on the condition that the local fitted surface is the searching region of PSO and the best average quality of the local triangles is the goal, the vertex position of the mesh is regulated. Finally, the threshold of the normal angle between the original vertex and regulated vertex is used to determine whether the vertex needs to be adjusted to preserve the detailed features of the mesh. Compared with existing methods, experimental results show that the proposed method can effectively improve the triangle quality of the mesh while preserving the geometric features and details of the original mesh.

## Introduction

Triangle mesh reconstruction based on physical measurement data is one of important researches in reverse engineering. There are often some non-standardization mesh cells in a reconstructed triangle mesh because of the complexities of the physical measurement data and the shortages of a mesh reconstruction algorithm, such as long-narrow triangles, holes, redundancy triangles and T-vertex triangles. When the finite element analysis is carried out on the physical model, the non-standardization mesh directly influences the precision, efficiency and convergence of the calculation result. In addition, the efficiency and stability of rapid prototyping manufacturing are also reduced because of the existence of the non-standardization mesh. Therefore, to obtain a high-quality mesh, it is necessary to standardize the mesh reconstructed by industrial computed tomography (ICT) images.

Because it is relatively easy to standardize a redundancy triangle and a T-vertex triangle, existing research on mesh standardization is based on two aspects, hole repairing and long-narrow triangle processing.

Hole repairing standardizes the mesh by filling the holes of the reconstructed triangle mesh. There are two main categories for the hole repairing method, repairing based on the mesh and repairing based on the volume data. Repairing based on the mesh partially fills the holes of the mesh without changing the original mesh topology, such as the algorithms of Zhao [1], Attene [2], Jun [3] and Panchetti [4]. Repairing based on the volume data [5–7] is divided into the following 3 steps: (1) converting the mesh reconstructed to the volume mesh; (2) filling the holes; and (3) reconstructing the triangle mesh by the extracting iso-surface for the volume mesh. The central contributions of the above-mentioned methods are triangulation of the holes and making a new triangle coordinate with the triangle area around the hole. Hence, the quality of the repaired triangle is not a serious consideration.

The standardization of a long-narrow triangle aims to achieve a regular triangle as much as possible by relocating the position of the triangle vertices. The Laplace standardization algorithm [8, 9] is popular and the most widely employed, and its key idea is to adjust the vertex position at a certain rate along the direction of the Laplace operator. The algorithm generally works quite well for improving the quality of the triangles. However, it can result in some shrinkage in the size of the mesh. Taubin [10] constructed a low-pass filter to effectively restrain the noise and mesh shrinkage, but it brought some disturbances. Vollmer [11] employed volume normalization for inhibiting the mesh shrinkage, yet the quality of the resulting mesh was general. Duguet [12] proposed an optimization algorithm combining the advantages of bilateral filtering and image filtering, and good mesh standardization was obtained. However, the error is bigger on the vertex curvature calculation. Chen [13] modified bilateral filtering and combined a quasi-Laplacian operator to relocate the vertex position. The algorithm can effectively make a uniform mesh while preserving the geometric detailed features of the original mesh. However, many parameters need to be set in the algorithm. There are many other methods for the long-narrow triangle standardization, such as those proposed by Zhu [14], Zheng [15], Wei [16], Gao [17], etc. These approaches of standardization improved the triangle quality of the mesh to some extent. However, the main considerations of the several approaches above preserved the geometric feature, reduced the volume shrinkage and improved the efficiency of the algorithm.

Each of the above-mentioned methods can only obtain good results in a specific aspect of mesh standardization. Some methods for mesh standardization are employed to obtain the uniform mesh, which can avoid the errors of finite element analysis. Some methods for mesh standardization are applied to get smaller error between standardized mesh and original mesh, which can reduce the reconstructed errors of CAD model. Some methods for mesh standardization are used to preserve the geometric features and details of the original mesh, which can improve the precision of finite element analysis. Some methods for mesh standardization are adopted to obtain the smoothed mesh, which can avoid stress concentration. However, methods described above cannot effectively improve the triangle quality of the mesh while preserving the geometric features and details of the original mesh, reducing the errors between standardized mesh and original mesh.

Because of the shortcoming of above-mentioned methods for mesh standardization, in this paper, we present a novel method for mesh standardization. First, a local curve surface for the vertex and its first-order adjacent vertices of the mesh is fitted using the least square method. Additionally, to improve the triangle quality of the mesh, we modify the PSO and apply it to achieve the optimum vertex on the fitted surface. Finally, threshold of the normal angle is used to determine whether the vertex needs to be regulated. The presented method can effectively improve the triangle quality of the mesh while preserving the geometric features and details of the original mesh.

The remainder of this paper is organized as follows. Section 2 describes a cubic curve surface fitting method for the vertex of the mesh and its adjacent vertices. Section 3 presents our PSO algorithm. Section 4 discusses the proposed method for mesh standardization. Section 5 shows some experimental results and analysis. The final section presents our conclusion.

## Local Curve Surface Fitting of the Mesh

Although the reconstructed triangle mesh is discrete, the mesh can be considered as continuous from a local perspective. Therefore, any one of the local meshes can be regarded as a local curve surface, and the vertices of the mesh are distributed on the surface. Hence, the standardization of the triangle is a procedure in which the triangle vertices of the mesh are regulated on the local surface.

References [18,19] provided an approach in which a vertex’s local surface is denoted by a cubic polynomial that fits the vertex of the mesh and its first-order adjacent vertices. Inspired by the method, in this paper, we employ the least squares to fit a cubic surface for the vertex and its adjacent vertices of the triangle mesh. To avoid geometry deformation and volume shrinkage of the reconstructed triangle mesh, the fitted surface is used for the constraint to the vertex adjustment. We regard vertex *V*_*i*_ as the origin of the coordinates. Consider the normal ***n***_***i***_ of *V*_*i*_ as the positive of the *z* axis, and create a local coordinate system to fit vertex *V*_*i*_ and its first-order adjacent vertices *N*(*V*_*i*_) into a cubic surface. The fitting curve surface is defined under the new coordinate system as:

$$f(x,y)=Ax^{3}+By^{3}+Cx^{2}y+Dxy^{2}+Ex^{2}+Fy^{2}+Gxy+Hx+Iy+J$$ (1)

The specific steps for solving the coefficient of the local surface equation (Eq 1) are as follows:

**Step 1** Solve the partial derivative of the curve surface equation *f*(*x*,*y*) and obtain the normal equation, *N*(*x*,*y*) of *f*(*x*,*y*):

$$\begin{array}{l} N(x,y)=(3Ax^{2}+2Cxy+Dy^{2}+2Ex+Gy+H,\\ \;3By^{2}+Cx^{2}+2Dxy+2Fy+Gx+I,-1) \end{array}$$ (2)

**Step 2** Set the normal vector of point (*x*_*i*_,*y*_*i*_,*z*_*i*_) as (*a*_*i*_,*b*_*i*_,*c*_*i*_). The normal (*a*_*i*_,*b*_*i*_,*c*_*i*_) is rewritten as (−*a*_*i*_/*c*_*i*_,−*b*_*i*_/*c*_*i*_,−1) in to be consistent with the form of Eq 2.

**Step 3** Write those coefficients of Eq 1 as a column vector, that is:

$$x={\left(A\;B\;C\;D\;E\;F\;G\;H\;I\;J\right)}^{T}$$ (3)

**Step 4** List the following equation for each point (*x*_*i*_,*y*_*i*_,*z*_*i*_) under the local coordinate system:

$$(x_{i}^{3}\;y_{i}^{3}\;x_{i}^{2}y_{i}\;x_{i}y_{i}^{2}\;x_{i}^{2}\;y_{i}^{2}\;x_{i}y_{i}\;x_{i}\;y_{i}\;1)x=z_{i}$$ (4)

$$({3x}_{i}^{2}\;0\;2x_{i}y_{i}\;y_{i}^{2}\;2x_{i}\;0\;y_{i}\;1\;0\;0)x=-a_{i}/c_{i}$$ (5)

$$({0\;3y}_{i}^{2}\;x_{i}^{2}\;2x_{i}y_{i}\;0\;y_{i}\;x_{i}\;0\;1\;0)x=-b_{i}/c_{i}$$ (6)

**Step 5** Combine Eqs 4, 5 and 6, and form a linear system of equations, that is:

Mx=b (7)

***M*** ∈ *R*^3*n*×10^ is a matrix, ***b*** ∈ *R*^3*n*×1^ is a column vector, and *n* represents the number of vertices *N*(*V*_*i*_). If *n* is less than 4, the nearer second-order adjacent vertices to *V*_*i*_ can be used.

**Step 6** Solve Eq 7, and obtain a feasible solution of the least square method, that is, the coefficients of the fitting surface equation *f*(*x*,*y*).

In mesh standardization of this paper, the fitted surface is used for the region constraint of PSO.

## Our PSO

### 3.1 Introduction of standard PSO

PSO [20,21] has been widely used in all types of engineering optimization problems because it is simple, easy to realize and has fewer adjustable parameters. The key idea of PSO is to find the optimal solution according to collaboration and information sharing between individuals in the group. The mathematical principle of PSO can be described as follows. In solution space, N particles present N possible solutions. The moving process of the particles is the searching process of the solution, rate of the particle stands for the searching direction, and each particle can determine the speed and position according to their own previous information and social information. The iterative formula for the speed and position of each particle can be defined as:

$$V_{i}^{t+1}=\omega V_{i}^{t}+c_{1}r_{1}(P_{i}^{t}-X_{i}^{t})+c_{2}r_{2}(P_{g}^{t}-X_{i}^{t})$$ (8)

$$X_{i}^{t+1}=X_{i}^{t}+V_{i}^{t+1}$$ (9)

The constraint of the particle speed can be presented as:

$$|V_{i}^{t+1}|\leq V_{max}$$ (10)

The individual previous optimal position of a particle can be calculated according to Eq 11:

$$P_{i}^{t+1}=\left\{ \begin{array}{l} X_{i}^{t+1},f(X_{i}^{t+1})\leq f(P_{i}^{t})\\ P_{i}^{t},\;f(X_{i}^{t+1})>f(P_{i}^{t}) \end{array}\right.$$ (11)

where $P_{i}^{t}\;and\;P_{g}^{t}$ are defined as:

$$P_{i}^{t}=\left\{X_{i}^{n}|min\{f(X_{i}^{1}),f(X_{i}^{2}),\cdots ,f(X_{i}^{t})\}\right\}$$ (12)

$$P_{g}^{t}=\left\{P_{i}^{t}|min\{f(P_{1}^{t}),f(P_{2}^{t}),\cdots ,f(P_{m}^{t})\}\right\}$$ (13)

where $V_{i}^{t+1}$ and $X_{i}^{t+1}$ represent the speed and position of the *i*-th particle carried out *t+*1 times, respectively; $V_{i}^{t}$ and $X_{i}^{t}$ stand for the speed and position of the *i*-th particle carried out *t* times, respectively; $P_{i}^{t}$ and $P_{i}^{t+1}$ denote the individual previous optimal solution of the *i-*th and the (*i+*1)-th iteration, respectively; $P_{g}^{t}$ is the group optimal solution after t times; *c*_1_ and *c*_2_ are learning factors; *m* is the population size; *ω* is a weight factor; *r*_1_ ∈ (0,1) and *r*_2_ ∈ (0,1) are the random distributions; *V*_*max*_ indicates the maximum limit speed of the particle; and *f*(∙) is a fitness function.

### 3.2 Modified PSO

Since PSO was proposed, it gains significant popularity and improvement. Shi *et al*. [22] introduced the inertia weight factor to constrict the velocity and better regulate the capability of search. Clerc *et al*. [23] presented an improved PSO with a constraint factor by analyzing the speed iterative equation of classical PSO. Clerc *et al*. found that introduction of constraint factor can prevent oscillation of particles, ensure the convergence of the algorithm, and analyzed it from the algebraic point of view (discrete time) and the analytical view (continuous time) respectively. Nowadays, the PSO with constraint factor becomes the canonical PSO algorithm because of good performance. Mendes *et al*. [24] proposed a fully informed PSO (FI-PSO) to improve the optimization performance. Liu *et al*. [25] addressed a novel PSO with the scale-free topologies and gave rise to a better balance between the convergence speed and the optimum quality. Motivated by the shortcoming of FI-PSO, Du *et al*. [26] proposed a PSO with limited information (LI-PSO), which provides adequate information of each particle yet avoids the waste of information. Liang et al. [27] presented a comprehensive learning PSO (CLPSO), which employs all other particles’ historical best information to update a particle’s velocity. This strategy preserves the diversity of the swarm and discourages premature convergence. Besides, there have been many improved PSO which focus on the model coefficients and the population structure [28–33].

However, Most of the existing PSO algorithms use the inertia factor depending on iterations, which thus ignores the effect of the error between fitness and the optimal solution. Besides, in most of PSO algorithms, each particle is usually influenced by the best particle among its neighborhood, which thus neglects some useful information from other neighbors. Promoted by above works, especially FI-PSO, LI-PSO, CLPSO and canonical PSO, we modify the PSO by introducing the center of particles and modifying the inertia factor.

First, standard PSO often falls into the local optimum, especially in high order optimization. To avoid the shortcomings of standard PSO, inspired by references [24, 26], we append a disturbance to the speed iteration formula (8) in this paper. The center of the current iterative particles is regarded as the disturbance, which indicates the influence of the most particles. The disturbance can effectively increase the information for particle searching optimization and disperse the appeal of the excellent particle to the whole particle swarm. The center of particles can be calculated by Eq 14.

$$P_{c}^{t}=\{X_{i}^{t}|Med\{f(X_{1}^{t}),f(X_{2}^{t}),\cdots ,f(X_{m}^{t})\}\}$$ (14)

where $P_{c}^{t}$ is the center of the particle swarm in the *t-*th iteration, $X_{i}^{t}$ is the position of *i-*th particle carrying out the *t-*th iteration, *f*(∙) is a fitness function, *Med*(∙) is a median filtering function, and *m* represents the population size.

Second, to adjust the searching region in real-time, we modify the inertia factor of PSO, which reflects the extent of inheritance to the speed of the particle itself. The search capability of PSO can be dynamically regulated by changing the value of *ω*. If *ω* is large, the PSO has strong global search capability; otherwise, the PSO has strong local search ability [22]. Linearly decreasing inertia weight (LDIW), as proposed by Shi [22,34], and its variants are widely employed at present. However, the value of *ω* depends only on the iterations under the strategy of LDIW, and the searching region of PSO cannot be adjusted according to the result of each calculation in real-time. To achieve a balance between global search and local search in PSO, in this paper, we apply the error between particle fitness and the optimal solution of the objective function to define an inertia factor, $\omega_{i}^{t}$ which can be adaptively regulated, that is:

$$\omega_{i}^{t}=\omega_{min}+(\omega_{max}-\omega_{min})(E_{i}^{t}(x)-{E(x)}_{min})/({E(x)}_{max}-{E(x)}_{min})$$ (15)

where *ω*_*min*_ and *ω*_*max*_ indicate the minimum and maximum of the inertia factor, respectively. After many experiments and calculations, Shi [34] found that the convergence speed and precision of the PSO are higher when *ω*_*min*_ = 0.4 and *ω*_*max*_ = 0.9. In this paper, the values of these two parameters are the same as the verified values by Shi. *E*(*x*)_*max*_ and *E*(*x*)_*min*_ represent, respectively, the maximum and minimum of the errors between the fitness of each particle and the optimal solution of the objective function in the process of iteration. $E_{i}^{t}(x)$ denotes the error between the fitness of the current particle carrying out the *t-*th iteration and the optimal solution of the objective function. Eq 15 shows that the value of the inertia factor, $\omega_{i}^{t}$, is proportional to the distance between the particle’s current position and the objective position. The global search ability and local search ability of the algorithm can be adjusted in real time in terms of the current position of the particle. Then, the solving precision of PSO is improved.

Finally, inspired by reference [23], to speed up the convergence rate, reduce the particle oscillation amplitude and avoid ineffective iteration, we introduce a constraint factor *ξ*. Because of the center of particles is added to the speed iterative equation described by Eq 17, the constraint factor is redefined as:

$$\xi =3/|3-\varphi -\sqrt{\varphi^{2}-6\varphi }|$$ (16)

where *φ* = *c*_1_ + *c*_2_ + *c*_3_ and *φ* > 6, *c*_1_, *c*_2_ and *c*_3_ are learning factors.

To sum up, PSO is modified by introducing the disturbance of the particles’ center, the constraint factor and the adaptive inertia factor. The speed formula and position formula of the modified PSO can be rewritten as:

$$V_{i}^{t+1}=\omega_{i}^{t}V_{i}^{t}+c_{1}r_{1}(P_{i}^{t}-X_{i}^{t})+c_{2}r_{2}(P_{g}^{t}-X_{i}^{t})+c_{3}r_{3}(P_{c}^{t}-X_{i}^{t})$$ (17)

$$X_{i}^{t+1}=X_{i}^{t}+\xi V_{i}^{t+1}$$ (18)

where $P_{c}^{t}$ is the center of the particle swarm in the *t-*th iteration, *c*_3_ is a center learning factor, and *r*_3_ ∈ (0,1) is a random distribution.

The performance comparisons among the modified PSO, classical PSO [22], canonical PSO [23] and comprehensive learning PSO [27] are particularly described in S1 Appendix.

In mesh standardization of this paper, the modified PSO is used to regulate the vertices of triangles on the fitted local curve surface and more triangles with good quality can be obtained. Here, the particles of PSO stand for various possible positions of the target vertex. Movements of the particles represent optimization process of the target vertex. And the best particle obtained by the PSO is the final position of the target vertex.

## Our Approach for Mesh Standardization

To quantitatively analyze the triangular standardization of the mesh, we have to determine an evaluation standard. In this paper, the triangle average quality [35] of the local mesh is used for the standard to evaluate the standardization of the mesh. The triangle average quality of the local mesh is defined as:

$$Q_{ave}=\sum_{k=1}^{T_{sum}}Q_{k}/T_{sum}$$ (19)

$$Q_{k}=4\sqrt{3}S_{k}/(l_{k1}^{2}+l_{k2}^{2}+l_{k3}^{2})$$ (20)

*T*_*sum*_ represents the number of triangles in the mesh; *Q*_*k*_ stands for the *k*-th triangle quality of the mesh; *S*_*k*_ denotes the *k*-th triangle area of the mesh; *l*_*k1*_, *l*_*k2*_, and *l*_*k3*_ indicate the edge lengths of the *k*-th triangle; and *Q*_*k*_ ∈ (0,1) describes the quality of a triangle. When *Q*_*k*_ = 1, the triangle is an equilateral triangle, whereas for smaller values of *Q*_*k*_, the triangle is longer-narrower in shape.

Therefore, the objective function of the proposed algorithm can be defined as:

$$minQ(N^{V_{i}})=1-Q_{ave}(N^{V_{i}})=1-(\sum_{k=1}^{T_{sum}}\frac{4\sqrt{3}S_{k}}{l_{k1}^{2}+l_{k2}^{2}+l_{k3}^{2}})/T_{sum}$$ (21)

where $N^{V_{i}}$ is a first-order neighborhood triangle set of vertex *V*_*i*_.

The constraints of vertex *V*_*i*_(*x*_*i*_,*y*_*i*_,*z*_*i*_) are:

$$\Delta x=max\left\{|x_{i}|-|x_{1}^{V_{i}}|,|x_{i}|-|x_{2}^{V_{i}}|,\cdots ,|x_{i}|-|x_{V_{sum}}^{V_{i}}|\right\}$$ (22)

$$\Delta y=max\left\{|y_{i}|-|y_{1}^{V_{i}}|,|y_{i}|-|y_{2}^{V_{i}}|,\cdots ,|y_{i}|-|y_{V_{sum}}^{V_{i}}|\right\}$$ (23)

$${\tilde{x}}_{i}\in \{x_{i}-\Delta x,x_{i}+\Delta x\}$$ (24)

$${\tilde{y}}_{i}\in \{y_{i}-\Delta y,y_{i}+\Delta y\}$$ (25)

$${\tilde{z}}_{i}=f({\tilde{x}}_{i},{\tilde{y}}_{i})=A{\tilde{x}}^{3}+B{\tilde{y}}^{3}+C{\tilde{x}}^{2}\tilde{y}+D\tilde{x}{\tilde{y}}^{2}+E{\tilde{x}}^{2}+F{\tilde{y}}^{2}+G\tilde{x}\tilde{y}+H\tilde{x}+I\tilde{y}+J$$ (26)

where ${\tilde{x}}_{i},{\tilde{y}}_{i},{\tilde{z}}_{i}$ are the new coordinate values of the vertex *V*_*i*_; *V*_*sum*_ indicates the number of the *V*_*i*_ first-order adjacent vertices; $x_{k}^{V_{i}},y_{k}^{V_{i}}\;(k=1,2,\cdots ,V_{sum})$ denote the *x*-value and *y*-value of the *k*-th vertex in the first-order adjacent vertices of *V*_*i*_, respectively; Δ*x*,Δ*y* stand for the range of the *x*-value and *y*-value when vertex *V*_*i*_ is regulated, respectively.

Eq 26 is a cubic surface equation, which is fitted by vertex *V*_*i*_ and its first-order adjacent vertices. The fitted surface is set as the constraint of *V*_*i*_ to ensure that the geometry size of the mesh does not shrink. In addition, to better preserve the detailed features of the mesh, the normal angle between the original vertex and the regulated vertex is used to determine whether the vertex needs to be adjusted when we have obtained the new position of the vertex using the improved PSO.

According to the above analyses, the presented approach about triangle mesh standardization can be implemented by the following steps.

**Step 1** Use Eq 1 to fit a local cubic surface for vertex *V*_*i*_ and its first-order adjacent vertices at the first.

**Step 2** Initialize the particles, and set the current position of the particles as individual previous optimal values. Determine the group’s optimal value using Eq 13, and calculate the center of particles according to Eq 14.

**Step 3** If the constraints of Eqs 24, 25 and 26 are satisfied, update the speed and position of each particle according to Eqs 17 and 18. Otherwise, *x* or *y* coordinate of the particle is assigned the boundary value according to Eqs 24 or 25, the opposite direction of the speed is set, and then calculate *z* coordinate of the particle according to Eq 26, and then update speed and position of the particle according to Eqs 17 and 18.

**Step 4** Calculate the fitness of each particle using Eq 21. Update the individual previous optimal positions and the group’s previous optimal position according to Eqs 12 and 13, and recalculate the center of particles using Eq 14.

**Step 5** If the end of the iteration or the error threshold meets the condition, go to the next step; otherwise, jump to step 3.

**Step 6** Store the new coordinate value of the vertex, and calculate and store the normal angle $\propto_{n_{i}}$ between the original vertex and the regulated vertex.

**Step 7** If $\propto_{n_{i}}$ is within the threshold range, update the vertex position and vertex normal vector information; otherwise, maintain the original position of the vertex.

**Step 8** If all vertices of mesh have been traveled, finish the mesh standardization; otherwise, go to step 1.

## Experimental Results and Discussions

The proposed algorithm is implemented in Visual C++6.0 and tested on a PC with an Intel Core2 2.2G Processor and 3G RAM. The parameter settings of the algorithm are shown in Table 1. We compare the standardization performance of the proposed method with several traditional methods, such as Laplace, Taubin, Vollmer and the state-of-the-art method of Chen, by making use of the triangle mesh reconstructed by ICT serial images. The values of the parameter of the compared methods are the same as the original references.

**Table 1:** Parameter settings of our algorithm.

Name	Value
iterations	50
population size	11
*c*_1_, *c*_2_, *c*_3_	2.05
error threshold	1.0e-4
normal angle threshold	8°

When the error of adjacent iterations is set to 1.0e-4, we can find the optimal position of the vertex after about 40 iterations according to the experimental results. Hence, the iterations of the proposed method can be set to 50. The population size, *m*, of PSO is generally set as 20~40, but it is worth noting that a larger value of *m* gives a lower efficiency of PSO. Furthermore, *m* is set as 11 because of the smaller region of the fitted surface. Here, *m* is set as an odd number to conveniently calculate the center of the particles. *c*_1_ is an individual learning factor denoting the cognitive ability of the particle itself; *c*_*2*_ is a global learning factor, which is used to indicate the information sharing cooperation ability of all particles; and *c*_3_ is a central learning factor standing for the balance ability of the center of the particles. Here, *c*_1_ = 2.05 and *c*_2_ = 2.05 are set empirically. *c*_3_ is also set as 2.05 to balance the influence among the center of the particles, the individual optimum and the global optimum. Reference [36] showed that the smaller the error threshold of the normal angle is, the better the preserving feature of mesh is and the worse the triangle quality of the mesh is. The error of the angle is commonly set as 5°∼15°, so we set the error threshold of the normal angle as 8° to strike a balance between the features and the triangle quality of mesh.

**Fig 1:**
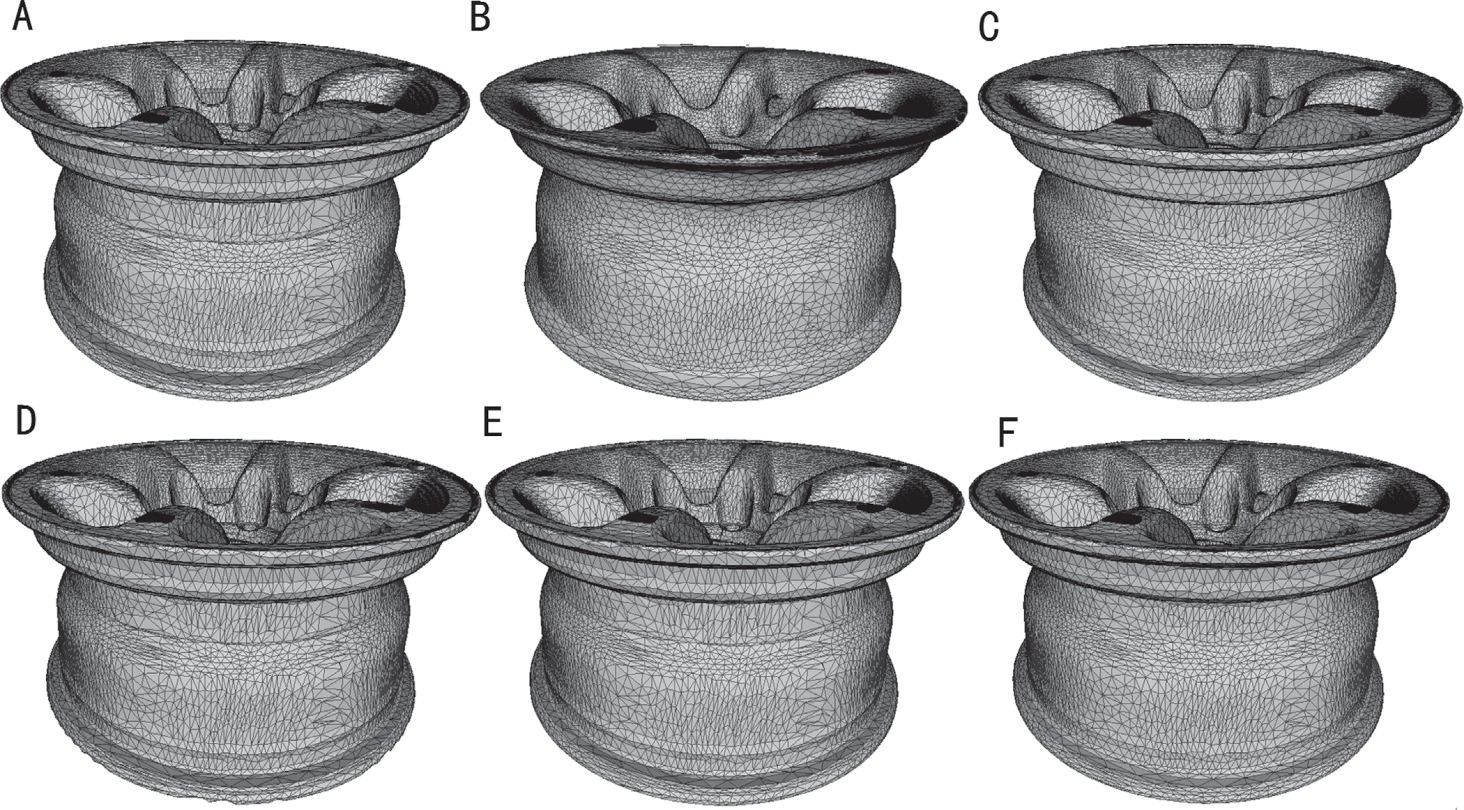
Comparison of the standardization algorithms on the wheel hub model. (A) original model; (B) standardization result by Laplace; (C) standardization result by Taubin; (D) standardization result by Vollmer; (E) standardization result by Chen; (F) standardization result by our method.

**Fig 2:**
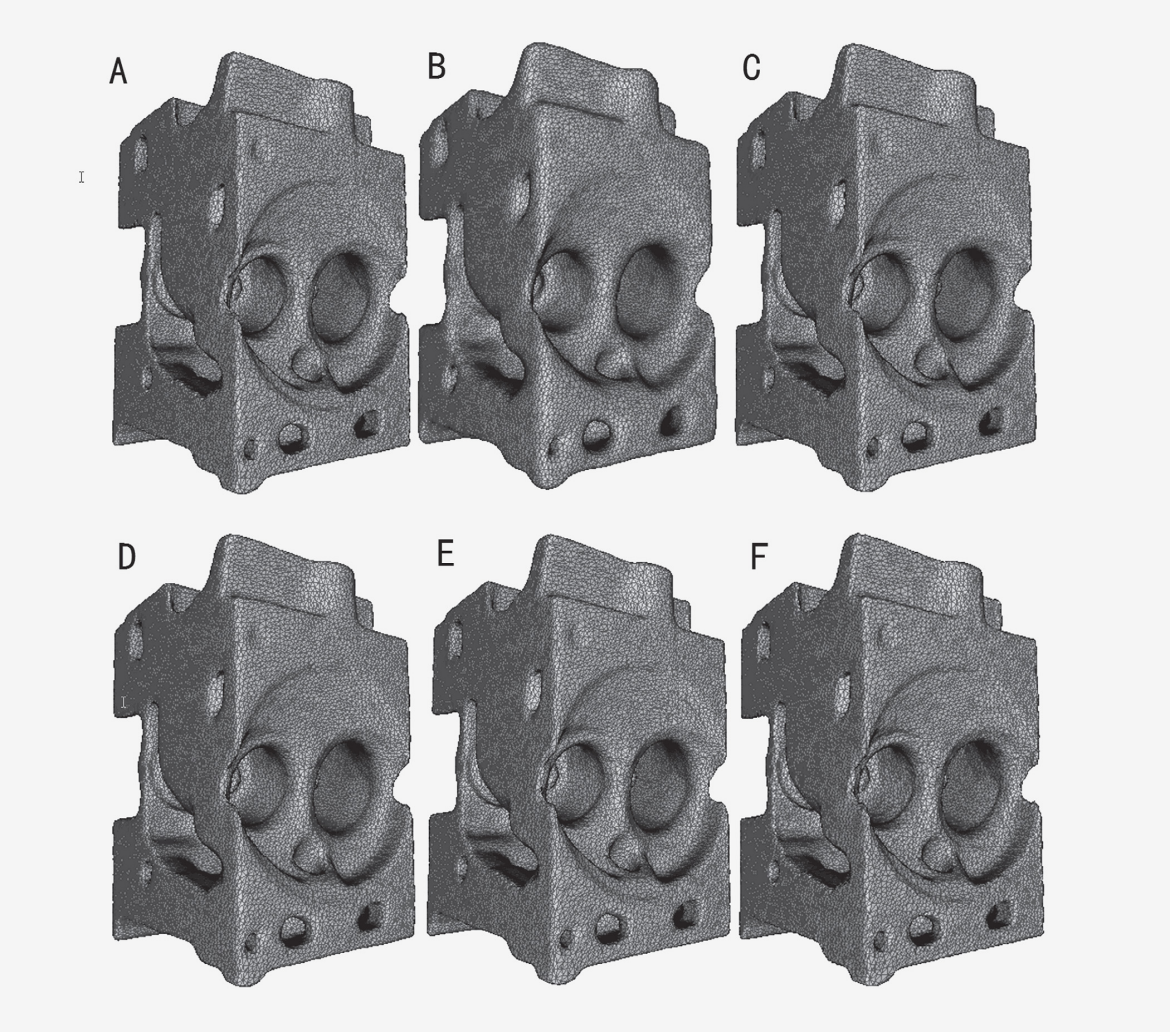
Comparison of the standardization algorithms on the cylinder head model. (A) original model; (B) standardization result by Laplace; (C) standardization result by Taubin; (D) standardization result by Vollmer; (E) standardization result by Chen; (F) standardization result by our method.

**Fig 3:**
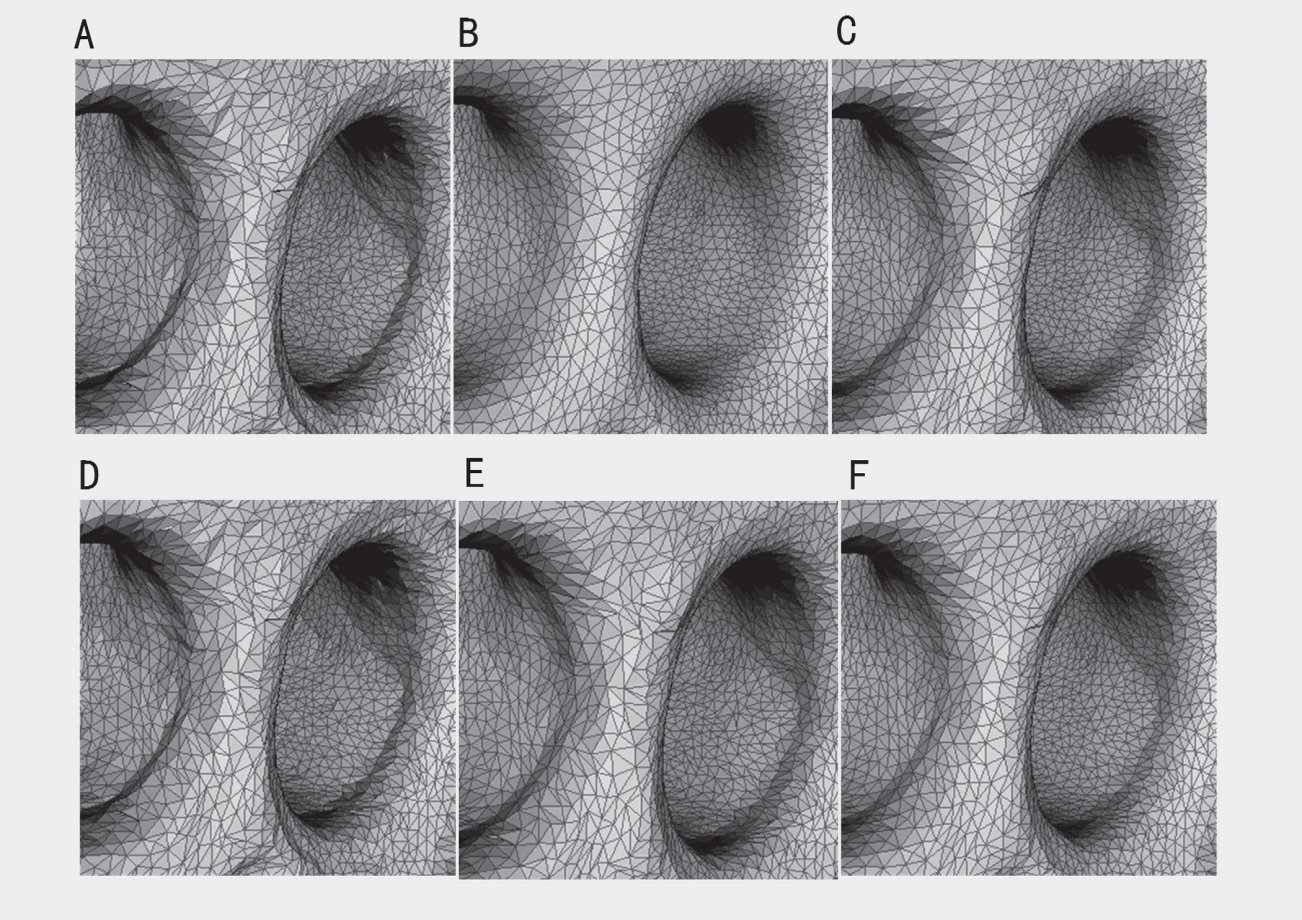
Zoomed region of the cylinder head model. (A) original model; (B) standardization result by Laplace; (C) standardization result by Taubin; (D) standardization result by Vollmer; (E) standardization result by Chen; (F) standardization result by our method.

The experimental results are shown in Figs 1–3. The comparisons of the standardization algorithms on the wheel hub model are shown in Fig 1. The quality of the mesh is better after Laplace standardization. However, some features of the mesh are lost, and the mesh is distorted (see Fig 1B). To some extent, the standardization results by Taubin and Vollmer preserve the feature of the mesh, whereas the triangle quality of the mesh is worse (see Fig 1C and 1D). The standardized mesh by Chen can nicely preserve the feature of the mesh, but the triangle quality of the mesh is general (see Fig 1E). The result of the proposed method shows that not only detailed features (e.g., wheel edges) of the mesh can be well preserved but that the triangle quality of the mesh is also better (see Fig 1F). Fig 2 shows the standardization results on the cylinder head model. The results of Taubin in Fig 2C, Chen in Fig 2E and the proposed method in Fig 2F can better preserve the feature of the mesh. However, the results of Laplace and Vollmer are greater distorted on the edge profile of the mesh (see Fig 2B and 2D). The results of the local zoomed region are as shown in Fig 3. The triangle is uniform, and the quality of the triangle is good after that the reconstructed mesh is standardized by the proposed method and the method of Laplace (see Fig 3B and 3F). However, many detailed features of the mesh standardized by Laplace’s method are lost (see Fig 3B), and the triangle quality of the mesh standardized by other methods are worse (see Fig 3C, 3D and 3E). Therefore, the proposed method can well standardize the mesh reconstructed by ICT serial images compared with existing methods.

To precisely compare the performances of the proposed and existing methods, the information of the mesh standardized by different methods is counted. Table 2 shows the related information of the original mesh. The bounding box size, surface area, volume, maximum error, average error of the meshes standardized by different methods and the consuming time of the different methods are shown in Table 3. Table 4 shows the triangle quality distribution of the different mesh standardization methods.

**Table 2:** Original data.

model	number of triangles	number of vertices	bounding box size /mm×mm×mm	area/mm^2^	volume/mm^3^
**wheel hub**	39355	19998	417.83×418.06×198.05	735373.81	3734234.00
**cylinder head**	1000084	50000	141.16×198.15×108.52	235250.42	1255598.13

**Table 3:** Performance comparison of different triangle mesh standardization methods.

model	method	bounding box size /mm×mm×mm	area/mm^2^	volume/mm^3^	maximum error/mm	average error/mm	time/s
**wheel hub**	Laplace	412.89×413.01×197.48	650562.44	3202049.75	7.1472	1.1144	0.235
	Taubin	417.67×417.98×198.00	722916.88	3732466.50	2.5570	0.1997	0.320
	Vollmer	416.59×416.84×197.87	703634.44	3546184.00	2.2813	0.3111	0.227
	Chen	417.90×418.31×198.05	730036.75	3731800.25	0.7679	0.0474	1.761
	Our	417.78×418.12×198.04	733668.25	3732216.28	0.8636	0.0372	2.746
**cylinder head**	Laplace	140.29×196.50×108.44	214356.06	1245656.00	2.6513	0.4138	0.718
		Taubin	140.90×198.21×108.74	233626.00	1255631.13	0.6379	0.0428	0.632
	Vollmer	140.52×197.55×108.50	228606.08	1228658.50	0.8444	0.1063	0.522
	Chen	141.03×198.30×108.84	234546.19	1253254.13	0.6460	0.0733	3.324
	Our	141.23×198.05×108.38	235146.37	1255243.26	0.5269	0.02956	5.643

**Table 4:** Triangle quality distribution of the different triangle mesh standardization methods.

method	Q ≤ 0.3		0.3 < *Q* ≤ 0.6		0.6 < *Q* ≤ 1.0	
	wheel hub	cylinder head	wheel hub	cylinder head	wheel hub	cylinder head
**Original model**	9.55%	7.78%	22.24%	15.89	68.21%	76.33%
**Laplace**	4.24%	3.82%	18.39%	14.45%	77.37%	81.73%
**Taubin**	10.84%	8.21%	26.53%	17.06%	62.62%	74.73%
**Vollmer**	12.62%	8.71%	23.88%	15.84%	63.50%	75.45%
**Chen**	6.76%	4.23%	20.13%	16.41%	73.11%	79.36%
**Our**	2.32%	1.01%	13.27%	10.76%	84.41%	88.23%

According to the data in Tables 2 and 3, for the wheel model, the error of bounding box size, surface area error and volume error of mesh standardized by Laplace are maximal. Error of bounding box size, surface area error and volume error of mesh standardized by Chen are much closer to original mesh. And our method nearly keeps the bounding box size, surface area and volume consistent with the original mesh’s. The maximum error and mean error of mesh standardized by Laplace are maximal. For the maximum error of the standardized mesh, Chen’s method is slightly less than the proposed method. However, the mean error of mesh standardized by the proposed method is minimal. For the cylinder head model, the errors of mesh standardized by Laplace are maximal, which contains the error of bounding box size, surface area error, volume error, maximum error and mean error. The errors of mesh standardized by Chen and Taubin are smaller than other methods except our method. According to the data in Table 4, compared with existing methods, the proposed method can obtain the largest number of triangles with good quality (0.6 < *Q* ≤ 1.0) and the least number of triangles with poor quality (Q ≤ 0.3). Where, the triangles with good quality on the wheel model is 84.41%, the triangles with good quality on the cylinder head model is 88.23%, the triangles with poor quality on the wheel model is 2.32%, the triangles with poor quality on the cylinder head model is 1.01%.

In conclusion, our method nearly keeps the bounding box size, the volume and the surface area of the mesh consistent with the original mesh’s, and better preserves the geometric features of the mesh. More importantly, the proposed method can sharply decrease the number of long-narrow triangles and greatly improve the quality of the triangles. However, we have to fit the curve surface according to the vertex and its first-order adjacent vertices in advance, and then search the optimal vertex. As a result, the consuming time of our method is relatively long.

## Conclusions

In this paper, we present a novel method for triangle mesh standardization based on the PSO. The proposed method can effectively improve the triangle quality of the mesh while preserving the geometric features and details of the original mesh. The PSO is modified by introducing the center of the particles, the constraint factor and the inertia factor, which can effectively avoid the local optimal, accelerate the convergence speed and adjust the searching region in real time. The fitted surface is used for the searching region of the PSO, which solves the volume shrinkage of the mesh standardized by most of existing algorithms. The threshold of the normal angle between the original vertex and the regulated vertex is used to determine whether the vertex needs to be regulated, to ensure that the detailed features of the triangle mesh are not lost. However, how to set the threshold of the normal angle in terms of the geometric feature of the triangle mesh and how to further shorten the consuming time of the proposed method are still problems worth considering.

## Supporting Information

S1 Appendix Performance comparisons of between the proposed PSO and the other PSO. (DOCX)
