# Supplementary material for: A Triangle Mesh Standardization Method Based on Particle Swarm Optimization
Source: PLoS One. 2016 Aug 10;11(8):e0160657. doi: 10.1371/journal.pone.0160657 (PMC4979957; doi:10.1371/journal.pone.0160657)
Supplement: S1 Appendix — (DOCX) [file pone.0160657.s001.docx]

**S1 Appendix**

**Performance comparisons of between the proposed PSO and the other PSO**

To compare with the classical PSO [22], canonical PSO [23] and comprehensive learning PSO [27], two frequently-used benchmark functions are employed. One is the Sphere function described by equation$f_{1}(x)$, and the other is the Rastrigrin function described by equation$f_{2}(x)$.

$f_{1}(x)=\sum_{i=1}^{n} x_{i}^{2}$ (s1)

$f_{2}(x)=\sum_{i=1}^{n} (x_{i}^{2}-10\cos(2\pi x_{i})+10)$ (s2)

Where, the value of parameters of the classical PSO ,canonical PSO and comprehensive learning PSO are the same as the original references. The initial value of the particles and the speed are some random distribution with zero mean and unit variance. The dimensions of benchmark functions are both 30 and the population size of PSO is 40.We can obtain the maximum, minimum and mean after the algorithms are run 30 times and the exact results are shown in table a1.

**Table a1．Performance comparisons of different PSO on benchmark functions**

| **function** | **global optimum** | **algorithm** | **dimensions** | **populations** | **iterations** | **min** | **max** | **mean** |
| --- | --- | --- | --- | --- | --- | --- | --- | --- |
| $f_{1}(x)$ | 0 | Classical PSO | 30 | 40 | 10000 | 6.287e-16 | 4.269e-05 | 4.481e-06 |
|  |  | Canonical PSO |  |  |  | 5.093e-26 | 8.131e-08 | 8.223e-09 |
|  |  | Our PSO |  |  |  | 4.002e-29 | 3.695e-09 | 3.750e-10 |
|  |  | CLPSO |  |  |  | 6.277e-11 | 4.458e-10 | 2.403e-10 |
| $f_{2}(x)$ | 0 | Classical PSO | 30 | 40 | 10000 | 1.492e+01 | 8.059e+01 | 3.870e+01 |
|  |  | Canonical PSO |  |  |  | 1.393e+01 | 6.368e+01 | 3.831e+01 |
|  |  | Our PSO |  |  |  | 6.913e+00 | 4.287e+01 | 1.701e+1 |
|  |  | CLPSO |  |  |  | 4.412e-05 | 4.864e-04 | 8.734e-05 |
| $f_{1}(x)$ | 0 | Classical PSO | 30 | 40 | 200000 | 7.128e-28 | 3.528e-13 | 3.613e-14 |
|  |  | Canonical PSO |  |  |  | 2.751e-148 | 7.038e-60 | 7.078e-61 |
|  |  | Our PSO |  |  |  | 1.480e-172 | 6.638e-105 | 7.639e-106 |
|  |  | CLPSO |  |  |  | 1.163e-130 | 1.965e-129 | 8.365e-130 |
| $f_{2}(x)$ | 0 | Classical PSO | 30 | 40 | 200000 | 73.969e+00 | 4.875e+01 | 4.363e+01 |
|  |  | Canonical PSO |  |  |  | 1.321e+01 | 6.251e+01 | 4.348e+01 |
|  |  | Our PSO |  |  |  | 4.231e-2 | 1.236e+01 | 3.403e-01 |
|  |  | CLPSO |  |  |  | 0 | 0 | 0 |

According to data in table a1, the proposed PSO is superior to the classical PSO and canonical PSO no matter whether it is 10000 iterations or 200000 iterations. The CLPSO is similar to our method on simple Sphere function, while CLPSO is more effective on multimodal Rastrigrin function.
